# Supplementary material for: TRIM28 SUMOylates and stabilizes NLRP3 to facilitate inflammasome activation
Source: Nat Commun. 2021 Aug 9;12:4794. doi: 10.1038/s41467-021-25033-4 (PMC8352945; doi:10.1038/s41467-021-25033-4)
Supplement: Supplementary file 1 — Supplementary Information [file 41467_2021_25033_MOESM1_ESM.pdf]

Supplementary Information for

TRIM28 SUMOylates and stabilizes NLRP3 to facilitate inflammasome activation

Ying Qin[1], Qi Li[1], Wenbo Liang[1], Rongzhen Yan[1], Li Tong[1], Mutian Jia[1], Chunyuan Zhao[1,2], and Wei Zhao[1,*]

[1]Department of Pathogenic Biology and Key Laboratory of Infection and Immunity of Shandong Province, School of Basic Medical Science, Cheeloo College of Medicine, Shandong University, Jinan, Shandong, China

[2]Department of Cell Biology, School of Basic Medical Science, Cheeloo College of Medicine, Shandong University, Jinan, Shandong, China

[*]Correspondence: wzhao@sdu.edu.cn (W.Z.)

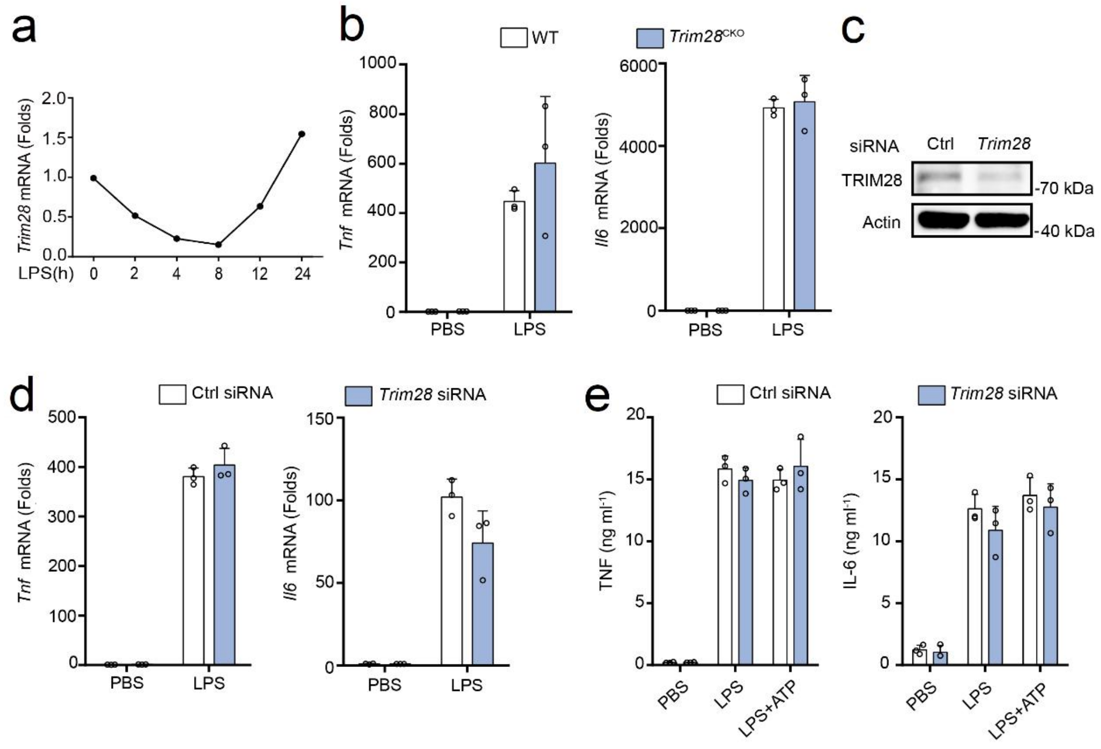

Supplementary Figure. 1 *Trim28* knockdown has no effects on NLRP3 inflammasome priming.

(**a**) RT-PCR analysis of *Trim28* mRNA in LPS-stimulated mouse PMs. (**b, d**) RT-PCR analysis of *Tnf* and *Il6* mRNA in LPS stimulated mouse PMs from WT or *Trim28*^{CKO} mice (**b**) or PMs transfected with Ctrl siRNA or *Trim28* siRNA (**d**). (**c**) Immunoblot analysis of TRIM28 expression in mouse PMs transfected with control (Ctrl) siRNA or *Trim28* siRNA for 48h. (**e**) ELISA analysis of TNF and IL-6 in supernatants from mouse PMs transfected with Ctrl siRNA or *Trim28* siRNA for 48h, following priming with LPS for 7 h and subsequent stimulation with ATP for 1h. All data are represented as means ± SD in b-d. Similar results were obtained from three independent experiments.

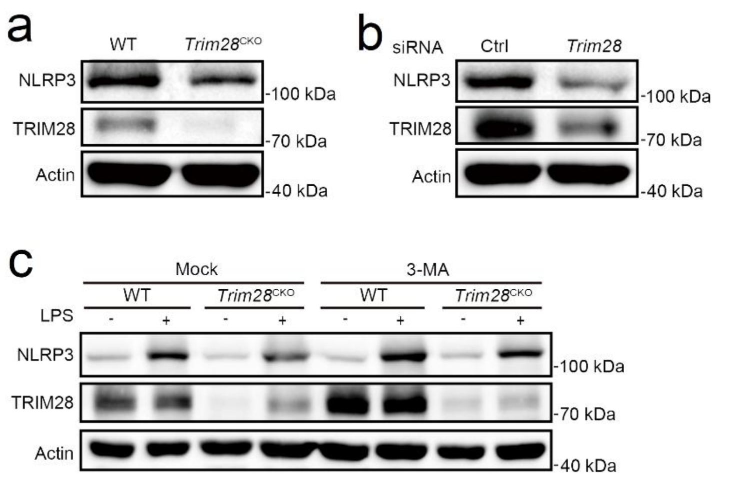

Supplementary Figure. 2 TRIM28 enhances NLRP3 expression.

(**a**, **b**) Immunoblot analysis of lysates of mouse PMs from WT or *Trim28*^{CKO} mice, or PMs transfected with Ctrl siRNA or *Trim28* siRNA for 48h. (**c**) Immunoblot analysis of NLRP3 expression in mouse PMs from WT or *Trim28*^{CKO} mice primed with LPS for 4h and then treated with 3-MA (10 mM) for 40min. Similar results were obtained from three independent experiments.

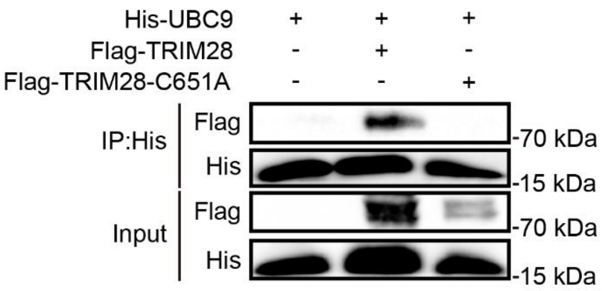

Supplementary Figure. 3 TRIM28 interacts with UBC9.

IP analysis of the interaction between UBC9 and TRIM28 in HEK293T cells transfected with His-UBC9, together with Flag-TRIM28 WT or C651A. Similar results were obtained from three independent experiments.

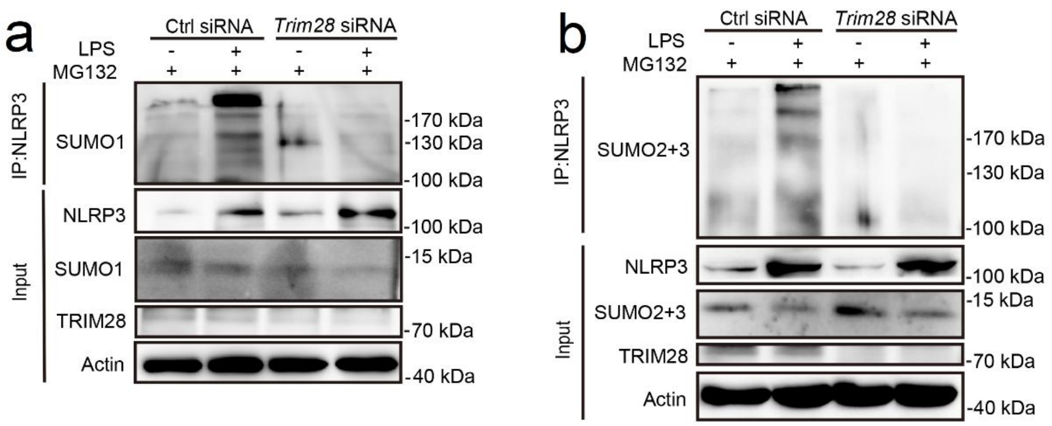

Supplementary Figure. 4 *Trim28* knockdown inhibits NLRP3 SUMOylation.

Immunoblot analysis of lysates from mouse peritoneal macrophages transfected with Ctrl siRNA or *Trim28* siRNAs for 48h, primed with LPS for 4h, together with MG132 (10 μM) treatment for 4h, and followed by SUMOylation assays with NLRP3 antibody. Similar results were obtained from three independent experiments.

Supplementary Table 1 Sequences of PCR primers used in this study

Name	Prime	Sequence
m*Actb*	Forward	5’-TGTTACCAACTGGGACGACA-3’
	Reverse	5’-CTGGGTCATCTTTTCACGGT- 3’
m*Nlrp3*	Forward	5'-TGGATGGGTTTGCTGGGAT-3'
	Reverse	5'-CTGCGTGTAGCGACTGTTGAG-3'
m*Il1b*	Forward	5'-ACCTTCCAGGATGAGGACATGA-3'
	Reverse	5'-AACGTCACACACCAGCAGGTTA-3'
m*Tnf*	Forward	5'-GCCACCACGCTCTTCTGTCT-3'
	Reverse	5'-TGAGGGTCTGGGCCATAGAAC-3'
m*Il6*	Forward	5'-ACAACCACGGCCTTCCCTAC-3'
	Reverse	5'-CATTTCCACGATTTCCCAGA-3'
m*Trim28*	Forward	5’- CACACTCACCTGCCGCGA-3’
	Reverse	5’-TCAACCTGCACTCGCTTCTG-3’
